# Facile preparation of alkali metal‐modified hollow nanotubular manganese‐based oxide catalysts and their excellent catalytic soot combustion performance

**DOI:** 10.1002/smo.20240022

**Published:** 2024-07-15

**Authors:** Chunlei Zhang, Siyu Gao, Xinyu Chen, Di Yu, Lanyi Wang, Xiaoqiang Fan, Ying Cheng, Xuehua Yu, Zhen Zhao

**Affiliations:** ^1^ State Key Laboratory of Heavy Oil Processing China University of Petroleum Beijing China; ^2^ Institute of Catalysis for Energy and Environment College of Chemistry and Chemical Engineering Shenyang Normal University Shenyang Liaoning China; ^3^ Department of Environmental Engineering Hebei University of Environmental Engineering Qinhuangdao Hebei China

**Keywords:** alkali metal, catalyst, centrifugal spinning, Mn‐based oxides, soot combustion

## Abstract

The soot emitted during the operation of diesel engine exhaust seriously threatens the human health and environment, so treating diesel engine exhaust is critical. At present, the most effective method for eliminating soot particles is post‐treatment technology. Preparation of economically viable and highly active soot combustion catalysts is a pivotal element of post‐treatment technology. In this study, different single‐metal oxide catalysts with fibrous structures and alkali metal‐modified hollow nanotubular Mn‐based oxide catalysts were synthesized using centrifugal spinning method. Activity evaluation results showed that the manganese oxide catalyst has the best catalytic activity among the prepared single‐metal oxide catalysts. Further research on alkali metal modification showed that doping alkali metals is beneficial for improving the oxidation state of manganese and generating a large number of reactive oxygen species. Combined with the structural effect brought by the hollow nanotube structure, the alkali metal‐modified Mn‐based oxide catalysts exhibit superior catalytic performance. Among them, the Cs‐modified Mn‐based oxide catalyst exhibits the best catalytic performance because of its rich active oxygen species, excellent NO oxidation ability, abundant Mn^4+^ ions (Mn^4+^/Mn^n+^ = 64.78%), and good redox ability. The *T*_10_, *T*_50_, *T*_90_, and CO_2_ selectivity of the Cs‐modified Mn‐based oxide catalyst were 267°C, 324°C, 360°C, and 97.8%, respectively.

## Graphical abstract

A series of alkali metal‐modified hollow nanotubular Mn‐based oxide catalysts with a special structure were prepared using centrifugal spinning. The prepared catalysts show excellent catalytic performance in catalytic soot combustion through active oxygen mechanism and nitrogen oxide‐assisted combustion mechanism, and have the potential for industrial practical application.

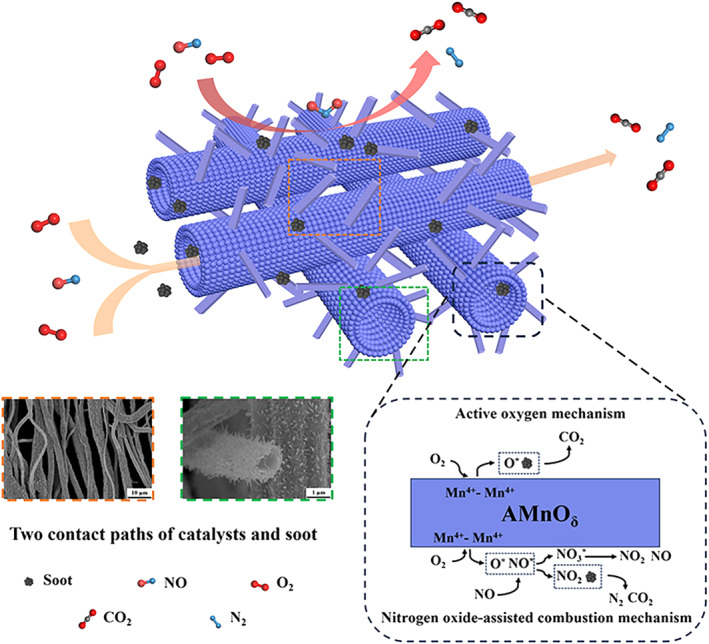

## 1 INTRODUCTION

Driven by the progress of contemporary society, the growth of vehicle ownership shows a significant trend. However, this growth is also accompanied by a significant increase in vehicle exhaust emissions, resulting in serious air pollution problems, which not only threaten biological health, but also have a significant adverse impact on the environment.[^1^, ^2^, ^3^] Among these pollutants, soot particles are the main pollutants in vehicle exhaust emissions and are the main cause of hazy weather.[^4^, ^5^] Soot particles can adsorb volatile organic compounds and pose serious hazards to the human respiratory, blood, and reproductive systems.[^6^, ^7^, ^8^] Hence, nations across the globe have implemented progressively rigorous policies to regulate vehicular particulate matter emissions. Currently, post‐treatment technology is widely acknowledged as the most efficacious approach for the elimination of soot. This technology can capture soot particles that have not yet entered the atmosphere using diesel particulate filters (DPFs) and relies on catalysts coated on the DPF surface to oxidize soot particles at low temperatures. Therefore, developing high‐performance soot catalysts is the key to advancing post‐treatment technology.[^9^, ^10^, ^11^] The catalytic performance in soot catalytic combustion primarily relies on the catalysts' intrinsic activity as well as the efficiency of contact between the soot and catalysts.[^12^, ^13^]

Up to now, researchers have synthesized various types of soot catalytic combustion catalysts that exhibit good intrinsic activity. For example, Pt, Au, Ag, and other noble metal catalysts have long lifespans and good low‐temperature catalytic activities.[^14^, ^15^] La‐based, Ce‐based, Sm‐contained, and other rare earth oxide catalysts have economical viability and resistance to toxicity.[^16^, ^17^, ^18^] Mn‐based, Fe‐based, Co‐based, and other transition metal oxide‐based catalysts have low prices and good redox properties.[^19^, ^20^, ^21^] Among them, transition metal and rare earth metal catalysts have garnered significant attention from researchers due to their good economy and strong redox ability. The particularity of the valence electron structure of manganese among transition metals makes it possible for it to form a variety of oxides with oxygen, and these various oxidation states can be interconverted. The variability and crystal structure of manganese ions in manganese oxides are beneficial in improving the catalytic performance of manganese oxides.[^22^, ^23^] Alkali metals are widely used in the modification of soot combustion catalysts; their melting characteristics at high temperatures lead to strong mobility of surface atoms, which is beneficial for the contact efficiency between the catalysts and soot. The alkalinity of alkali metals is beneficial to adsorb NO and generate two nitrogen‐containing compounds (nitrogen dioxide and nitrate species). Nitrogen dioxide can desorb from the catalyst surface and directly oxidize soot, while NO_3_^−^ and Mn^n+^/alkali metals form nitrate species. As temperature increases, nitrate species decompose into NO/NO_2_, which promotes soot combustion.[^24^, ^25^, ^26^]

In addition, the synthesis of catalysts with special morphologies can effectively enhance the contact efficiency between soot and catalysts, thereby achieving the goal of optimizing the performance of the catalyst.[^27^, ^28^, ^29^] Nanofibers, as one‐dimensional nanostructures, possess advantages including the high porosity and specific surface area, which are beneficial for the capture of soot. Therefore, they are considered to be the most promising catalysts.[^30^, ^31^, ^32^, ^33^] At present, hollow nanotubular catalysts are typically prepared using electrospinning,[^34^, ^35^] the hydrothermal method,[^36^, ^37^] template method,[^38^, ^39^] or other methods. However, widespread application is difficult because of the high cost, high energy consumption, and low efficiency. Hence, this study adopted an alternative process to prepare hollow nanotube structure catalysts, namely the centrifugal spinning method. This method uses the centrifugal force generated by a rotating shaft to eject the prepared precursor sol through small holes located on the side of the centrifuge. The precursor was exposed to the air, solidified or evaporated by the solvent, and finally, dry nanofibers were formed and collected. The preparation method has not only a low cost and simple operation scheme but also can prepare many different types of materials through the modulation of the precursor composition, and has high production efficiency.

Based on the above discussion, a series of different single‐metal oxide catalysts with fibrous structures were prepared using centrifugal spinning method, and hollow nanotubular manganese oxide catalysts were modified with different alkali metals. The physicochemical properties and catalytic performance of the prepared catalysts were characterized and evaluated. On this basis, the impact of the synergistic effect between the morphology and active components of the catalyst on its catalytic performance is proposed, and a discussion on the reaction mechanism of this type of catalyst is conducted.

## 2 EXPERIMENTAL SECTION

### 2.1 Catalyst preparation

#### 2.1.1 Preparation of different single‐metal oxide catalyst

Different single‐metal oxide catalysts with fibrous structures were prepared using the centrifugal spinning method. Set amounts of metal acetate or metal nitrate were dissolved in a homogeneous mixture of ethanol and water, respectively. After adding a small amount of CH_3_COOH, a set amount of PVP (Kw = 1,300,000) was dissolved in the above solution, and a homogeneous sol precursor was obtained by magnetic stirring at 50°C for 30 min. A commercial centrifugal spinning machine was used to centrifuge the precursor sol at 6000 rpm. The collected fibers were dried and then calcined in an air atmosphere. Heat up at a rate of 2°C·min^−1^ to 300°C, and after 1 h, rapidly increase the temperature to 500°C within 20 min. After 6 h at 500°C, the required single metal oxide catalyst was finally synthesized. The expression of catalysts and raw material consumption are shown in Table S1.

#### 2.1.2 Preparation of alkali metal‐modified hollow nanotubular Mn‐based oxide catalysts

Alkali metal acetate and manganese acetate tetrahydrate with a molar ratio of 0.3:1 were dissolved in a homogeneous mixture of ethanol and water. After adding a small amount of CH_3_COOH, a set amount of PVP (Kw = 1,300,000) was dissolved in the above solution, and a homogeneous sol precursor was obtained by magnetic stirring at 50°C for 30 min. The subsequent centrifugation, drying, and calcination processes were the same as those in Section 2.1.1. Finally, different alkali metal‐modified hollow nanotubular Mn‐based oxide catalysts were obtained. The expression of catalysts and raw material consumption are shown in Table S2.

### 2.2 Physical and chemical characterization

The physicochemical properties of the catalysts were characterized using various techniques including XRD, BET, SEM, H_2_‐TPR, Soot‐TPR,O_2_‐TPD, XPS, NO‐TPO, and in situ DRIFTS. The detailed characterization procedures are stated in the Supporting Information.

### 2.3 Activity measurements

The activity, stability, and sulfur resistance of the catalysts were evaluated by temperature programmed oxidation. The detailed evaluation procedures are contained in the Supporting Information.

## 3 RESULTS AND DISCUSSION

### 3.1 Structural features of the as‐prepared catalysts

#### 3.1.1 XRD patterns of the as‐prepared catalysts

**FIGURE 1:**
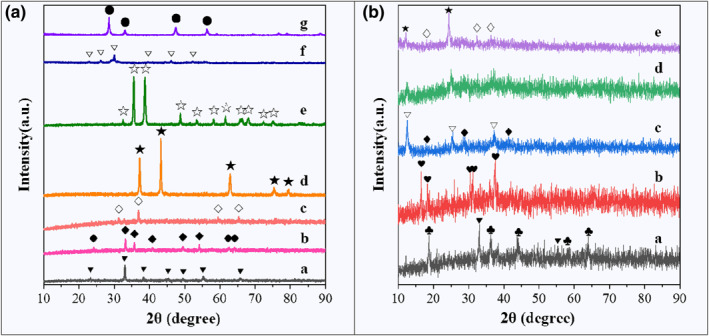
XRD patterns of different single metal oxide catalysts with fibrous shape (a); XRD patterns of alkali metal‐modified hollow nanotubular Mn‐based oxide catalysts (b). ((a): a: Mn_2_O_3_; b: Fe_2_O_3_; c: Co_3_O_4_; d: NiO; e: CuO; f: La_2_O_3_; g: CeO_2_; (b): a: Li_0.3_MnO_δ_; b: Na_0.3_MnO_δ_; c: K_0.3_MnO_δ_; d: Rb_0.3_MnO_δ_; e: Cs_0.3_MnO_δ_).

The XRD patterns of different single‐metal oxide catalysts with fibrous structures are shown in Figure 1a, and all catalysts show their common metal oxide crystal phases. For the Mn_2_O_3_ catalyst, the diffraction peaks at 2*θ* = 23.3°, 33.0°, 38.4°, 45.2°, 49.4°, 55.2°, and 65.8° belong to the Mn_2_O_3_ crystal form (labeled as ‘▼’, JCPDS PDF# 41‐1442).^[40]^ The feature peaks of the Fe_2_O_3_ catalyst, which are observed at 2θ of 24.3°, 33.2°, 35.6°, 40.9°, 49.5°, 54.1°, 62.5°, and 64.2° can be assigned to Fe_2_O_3_ (labeled as ‘◆’, JCPDS PDF# 33‐0664).^[41]^ For the Co_3_O_4_ catalyst, the peaks at 2θ of 31.3°, 36.9°, 59.4°, and 65.3° are in good agreement with the Co_3_O_4_ crystal form (labeled as ‘◇’, JCPDS PDF# 43‐1003).^[42]^ The diffraction peaks at 2*θ* = 37.1°, 43.2°, 62.9°, 75.4°, and 79.4° of the NiO catalyst corresponded to NiO (labeled as ‘★’, JCPDS PDF# 47‐1049).^[43]^ The diffraction peaks of the CuO catalyst are located at 2*θ* = 32.5°, 35.5°, 38.7°, 48.8°, 53.4°, 58.2°, 61.6°, 66.2°, 68.0°, 72.4°, and 75.0°, which are highly consistent with the characteristic peaks of a CuO phase (labeled as ‘☆’, JCPDS PDF# 45‐0937).^[44]^ For the La_2_O_3_ catalyst, the diffraction peaks, which are located at 2θ of 26.1°, 29.1°, 30.0°, 39.5°, 46.1°, and 52.1°, are attributed to La_2_O_3_ (labeled as ‘▽’, JCPDS PDF# 05‐0602).^[45]^ The diffraction peaks, marked by ‘●’ of the CeO_2_ catalyst at 2θ of 28.5°, 33.0°, 47.5°, and 56.3°, correspond to the CeO_2_ crystal form (JCPDS PDF# 34‐0394).^[46]^

The XRD patterns of alkali metal‐modified hollow nanotubular Mn‐based oxide catalysts are shown in Figure 1b. The modification of different alkali metals has a significant effect on the crystal form and crystallinity of Mn‐based catalysts. There are two crystal forms of the Li_0.3_MnO_δ_ catalyst, which are the Mn_2_O_3_ crystal form (corresponding diffraction peaks at 2*θ* = 33.0°, 55.2°, labeled as ‘▼’, JCPDS PDF# 41‐1442) and the spinel LiMn_2_O_4_ crystal form (corresponding diffraction peaks at 2*θ* = 18.6°, 36.1°, 43.9°, 58.1°, and 63.8°, labeled as ‘♣’, JCPDS PDF# 35‐0782).^[47]^ The diffraction peaks of the Na_0.3_MnO_δ_ catalyst are located at 2*θ* = 16.5°, 18.2°, 30.4°, 31.2°, and 37.3°, which are highly consistent with the characteristic diffraction peaks of the Na_2_Mn_5_O_10_ crystal form (labeled as ‘♥’, JCPDS PDF# 27‐0749).^[48]^ The K_0.3_MnO_δ_ catalyst also has two crystal forms, the cryptomelane type K_2‐x_Mn_8_O_16_ crystal form (corresponding diffraction peaks at 2*θ* = 18.3°, 28.9°, and 41.4°, labeled as ‘♦’, JCPDS PDF# 44‐1386) and the birnessite type K_2_Mn_4_O_8_ crystal form (corresponding diffraction peaks at 2*θ* = 12.6°, 25.4°, and 37.3°, labeled as ‘▽’, JCPDS PDF# 16‐0205).[^49^, ^50^] The Rb_0.3_MnO_δ_ catalyst is a rubidium–manganese composite metal oxide. The Cs_0.3_MnO_δ_ catalyst is a composite crystal of Cs_4_Mn_14_O_27_·xH_2_O (corresponding diffraction peaks at 2*θ* = 12.2° and 24.5°, labeled as ‘★’, JCPDS PDF# 50‐0010) and Mn_3_O_4_ (corresponding diffraction peaks at 2*θ* = 18.0°, 32.4°, and 36.1°, labeled as ‘♢’, JCPDS PDF# 18‐0803).[^51^, ^52^]

#### 3.1.2 SEM images of the as‐prepared catalysts

**FIGURE 2:**
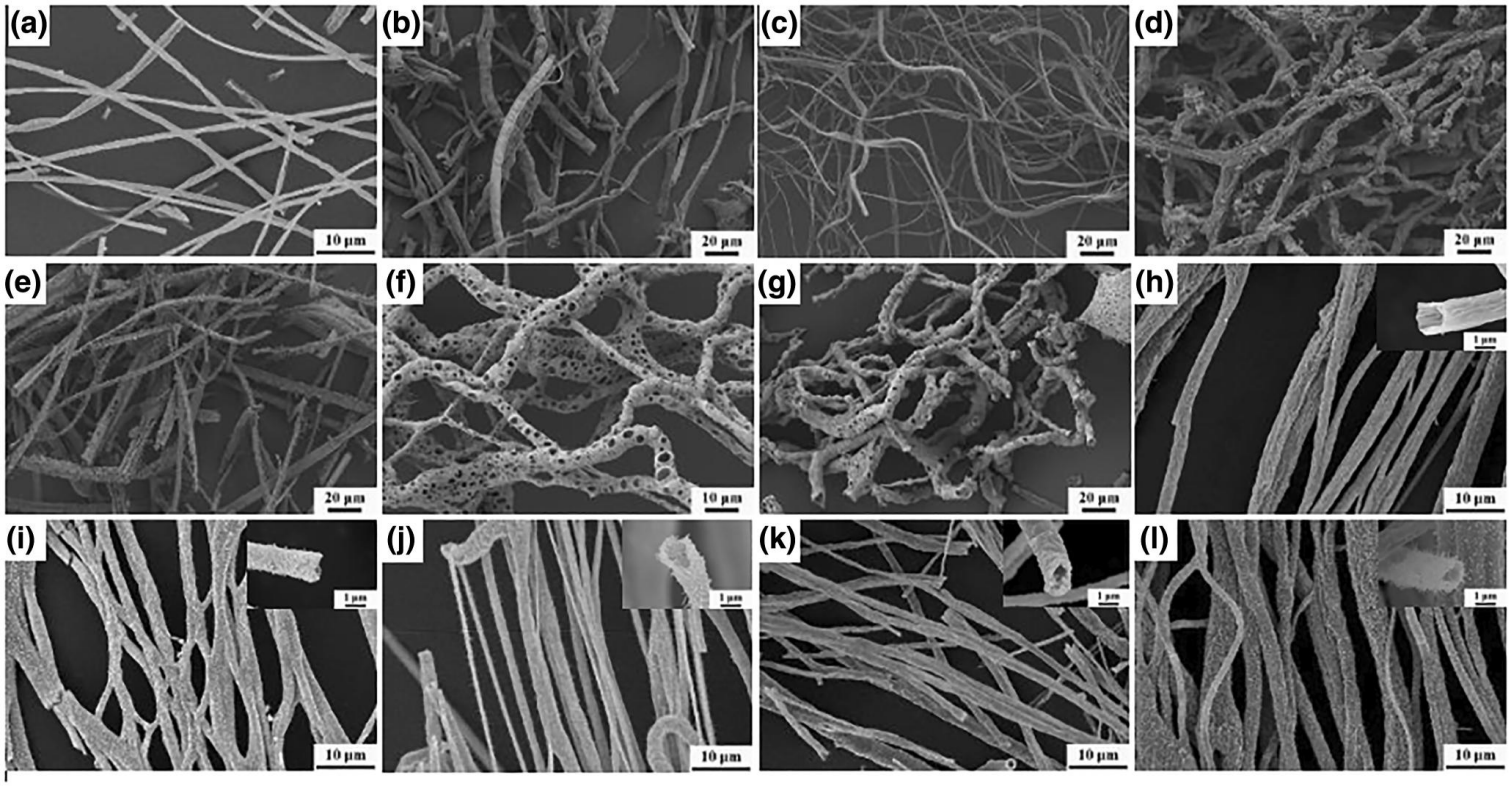
SEM images of as as‐prepared catalysts. (a) Mn_2_O_3_; (b) Fe_2_O_3_; (c) Co_3_O_4_; (d) NiO; (e) CuO; (f) La_2_O_3_; (g) CeO_2_; (h) Li_0.3_MnO_δ_; (i) Na_0.3_MnO_δ_; (j) K_0.3_MnO_δ_; (k) Rb_0.3_MnO_δ_; (l) Cs_0.3_MnO_δ_.

The morphology of the catalysts was studied using SEM. The SEM images of different single‐metal oxide catalysts are shown in Figure 2a–g. All single‐metal oxide catalysts have fibrous structures. The Mn_2_O_3_, Fe_2_O_3_, Co_3_O_4_, NiO, and CuO catalysts were hollow nanotubular structures, while the La_2_O_3_ and CeO_2_ catalysts showed porous fibrous structures. The specific morphologies of various catalysts differ slightly. The surfaces of Mn_2_O_3_, Fe_2_O_3_, and Co_3_O_4_ catalyst fibers were smooth, and the average diameters of the fibers were 1.22, 5.79, and 1.26 μm, respectively. The surfaces of the NiO and CuO catalysts were relatively rough, and the average fiber diameters were 3.08 and 4.43 μm, respectively. The La_2_O_3_ and CeO_2_ catalysts have porous fiber structures, and a large number of pore structures are evenly distributed on the fiber surface. The pores of the La_2_O_3_ catalyst were significantly more abundant than those of the CeO_2_ catalyst, and the average diameters of the two catalysts were 3.90 and 5.26 μm, respectively.

Figure 2h–l shows the SEM images of the alkali metal‐modified hollow nanotubular Mn‐based oxide catalysts. The prepared catalysts possess hollow nanotubular structures, but modification with different alkali metals makes the fibers exhibit different morphologies. The surface of the Li_0.3_MnO_δ_ catalyst appears wrinkled, with an average diameter of roughly 2.11 μm. The tubular structure of the Na_0.3_MnO_δ_ catalyst with an average diameter of roughly 1.75 μm forms via the stacking of nanorods. The average diameter of K_0.3_MnO_δ_ nanotube catalyst is approximately 1.7 μm, and a large number of uniformly distributed nanoneedle structures can be observed on the surface of the nanotubes, resembling cactus palms (Figure 2j). The tubular structure of the Rb_0.3_MnO_δ_ catalyst with a diameter of approximately 1.65 μm comprises stacked nanosheets of particles. The average diameter of the Cs_0.3_MnO_δ_ catalyst is approximately 1.76 μm, the morphological resemblance to the K_0.3_MnO_δ_ catalyst is observed, yet the nanoneedle formations exhibiting a uniform distribution across the nanotube surfaces are discernibly more dense (Figure 2l).

#### 3.1.3 N_2_ adsorption–desorption isotherms of the as‐prepared catalysts

To investigate the texture properties of the prepared catalysts, the N_2_ adsorption–desorption isotherms were recorded (Figure S1). As shown in Figure S1A,C, the prepared catalysts showed Type‐IV isotherms, and a hysteresis loop composed of inclined adsorption and desorption curves was observed. The hysteresis loop type was an H3 type, indicating that the pore structure of the catalysts formed via particle accumulation, producing an irregular slit structure. As shown in Table S3, except for the La_2_O_3_ and CeO_2_ catalysts, the specific surface areas of the other catalysts were similar, and the specific surface areas were in the range of 1.8–14.0 m^2^·g^−1^. In contrast, La_2_O_3_ and CeO_2_ catalysts are slightly larger, which is attributable to the emergence of numerous pore structures on the fiber surface.

### 3.2 Redox properties of the as‐prepared catalysts

#### 3.2.1 H_2_‐TPR, O_2_‐TPD, and soot‐TPR results of the as‐prepared catalysts

**FIGURE 3:**
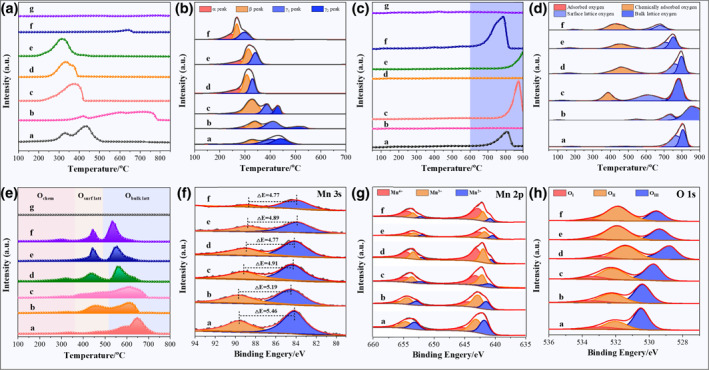
H_2_‐TPR (a) and O_2_‐TPD (c) curves of different single metal oxide catalysts with fibrous shape (a: Mn_2_O_3_; b: Fe_2_O_3_; c: Co_3_O_4_; d: NiO; e: CuO; f: La_2_O_3_; g: CeO_2_); H_2_‐TPR (b), O_2_‐TPD (d) and Soot‐TPR (e) curves of alkali metal‐modified hollow nanotubular Mn‐based oxide catalysts; The Mn 3s (f), Mn 2p (g) and O 1s (h) XPS spectra of alkali metal‐modified hollow nanotubular Mn‐based oxide catalysts (a: Mn_2_O_3_; b: Li_0.3_MnO_δ_; c: Na_0.3_MnO_δ_; d: K_0.3_MnO_δ_; e: Rb_0.3_MnO_δ_; f: Cs_0.3_MnO_δ_; g: Without catalyst).

Catalytic soot combustion involves multiphase deep oxidation reactions, and the oxidation ability of the catalyst plays a crucial role in the catalytic combustion. Consequently, the oxidation performance of the prepared catalysts was characterized by H_2_‐TPR. Figure 3a shows the H_2_‐TPR reduction curve of different single‐metal oxide catalysts with fibrous structures. The reduction curve of the Mn_2_O_3_ catalyst corresponds to a continuous reduction process of Mn^4+^→Mn^3+^→Mn^2+^ in a hydrogen atmosphere. The reduction peak of the Fe_2_O_3_ catalyst corresponds to the reduction of ferric oxide to ferroferric oxide and then elemental Fe. The reduction curve of the Co_3_O_4_ catalyst corresponds to the process of reducing trivalent cobalt ions to cobalt elemental. The reduction peak of the NiO catalyst corresponds to the reduction of NiO. The reduction curve of the CuO catalyst corresponds to the process of reducing CuO to elemental Cu.

The H_2_‐TPR curves of alkali metal‐modified hollow nanotubular Mn‐based oxide catalysts are shown in Figure 3b. The peak shape and peak position of H_2_‐TPR curves obtained for different catalysts differ significantly, indicating that modification with different alkali metals for Mn‐based catalysts introduces various redox properties. All H_2_‐TPR curves of alkali metal‐modified Mn‐based catalysts exhibit three distinct reduction peaks, including an α, β, and *γ* peak. The *α* reduction peak can be assigned to the reduction of surface oxygen species, the *β* reduction peak can be assigned to the reduction of Mn^4+^ to Mn^3+^, and the *γ*_1_ and *γ*_2_ reduction peaks correspond to the reduction of surface and bulk Mn^3+^ to Mn^2+^, respectively.^[53]^ Different alkali metals also exhibit significant effects on the reduction peak temperature of catalysts. Among them, the reduction peak temperature of the Cs_0.3_MnO_δ_ catalyst is the lowest, indicating that it has the best redox performance.

Active oxygen species are key active species involved in catalytic combustion reactions, which not only directly oxidize soot but also participate in forming NO_2_. Therefore, the oxygen species in the catalyst were detected and analyzed using O_2_‐TPD. It can be observed from Figure 3c that the curves of all single‐metal oxide catalysts are flat in the low‐temperature and medium‐temperature regions; only some catalysts have higher lattice oxygen desorption within the temperature regime exceeding 600°C. This phenomenon indicated that the single‐metal oxide catalysts possess few active oxygen species and cannot be detected using O_2_‐TPD. The O_2_‐TPD curves (Figure 3d) of the alkali metal‐modified Mn‐based oxide catalysts exhibit four categories of oxygen desorption peaks, which are attributed to the desorption of physical adsorption oxygen, chemical adsorption oxygen, surface lattice oxygen, and bulk lattice oxygen, respectively.^[54]^ Since the catalytic burnout temperature of soot is lower than 450°C by as‐prepared catalysts, the chemisorbed oxygen species are the primary reactive oxygen species. Compared with the Mn_2_O_3_ catalyst, the amount of chemisorbed oxygens for the Li_0.3_MnO_δ_ catalyst was not increased significantly, but the amount of chemisorbed oxygens for Na_0.3_MnO_δ_, K_0.3_MnO_δ_, Rb_0.3_MnO_δ_, and Cs_0.3_MnO_δ_ were obviously increased. The chemisorbed oxygen desorption peaks of the Na_0.3_MnO_δ_ and Cs_0.3_MnO_δ_ catalysts appeared at lower temperatures. In addition, the chemisorbed oxygen desorption peak area of the Cs_0.3_MnO_δ_ catalyst was the largest among the alkali metal‐modified Mn‐based oxide catalysts. This phenomenon indicates that alkali metal doping is highly beneficial for adsorbing chemical oxygen species.

The catalysts were also characterized using soot‐TPR with soot as the reducing agent in Ar atmosphere. To better verify the role of catalysts in soot‐TPR characterization, the characterization results of pure soot without catalyst under the same test conditions were used as the contrast results (Figure 3e (g)). Under the condition of no catalyst, the curve of soot‐TPR was flat, indicating that there was basically no CO_2_ generation. After adding the catalysts, as the temperature increases, the oxygen species of the catalysts successively reacted with soot and formed CO_2_, as shown in Figure 3e (a–f). The soot‐TPR curves can be clearly delineated into three types of peaks, which are attributable to the redox reaction of three distinct oxygen species with soot. In the low‐temperature regime, the oxygen species is ascribed to chemisorbed oxygen, while in the medium‐temperature range, it is associated with surface lattice oxygen. Furthermore, in the high‐temperature zone, the oxygen species is attributed to bulk lattice oxygen.^[55]^ Because soot can burn out before 450°C under the action of these catalysts, The chemisorbed oxygen species and surface lattice oxygen species have a major contribution to the catalytic soot combustion. In the soot‐TPR curve of Figure 3e, the K_0.3_MnO_δ_ and Cs_0.3_MnO_δ_ catalysts have more chemisorbed oxygen species in the low‐temperature region. This is consistent with the outcomes of O_2_‐TPD characterization, indicating that they possess more reactive oxygen species in the catalytic process.

#### 3.2.2 XPS spectra of the as‐prepared catalysts

As depicted in Figure 3g, the overlapping binding energies exhibited by the three oxidation states of manganese ions (Mn^4+^, Mn^3+^, and Mn^2+^) and the broadening of the peak caused by spin multiplicity render it difficult to accurately analyze the oxidation state of Mn‐based only on the Mn 2p spectrum. Notably, since the binding energy difference (Δ*E*) of the Mn 3s spin‐orbit doublet state exhibits a distinct correlation with the average Mn oxidation state (AOS), which can be expressed as: AOS = 8.956−1.126×Δ*E*, the AOS of the Mn in the catalyst can be accurately determined by observing the Δ*E* value.^[56]^ As shown in Figure 3f, compared with the Δ*E* = 5.46 of the Mn_2_O_3_ catalyst, the Δ*E* values of the other catalysts modified with alkali metals were reduced significantly, indicating that the AOS of Mn in the Mn‐based oxide catalysts modified with alkali metals was improved significantly. The AOS values of MnO_δ_, Li_0.3_MnO_δ_, Na_0.3_MnO_δ_, K_0.3_MnO_δ_, Rb_0.3_MnO_δ_ and Cs_0.3_MnO_δ_ catalysts were 2.81, 3.11, 3.43, 3.58, 3.45 and 3.58, respectively. Among them, the Cs_0.3_MnO_δ_ catalyst has the highest oxidation state, that is, the highest Mn^4+^ abundance, consistent with the characterization results of the Mn 2p spectrum. As shown in Table 1, the Mn^4+^ abundance is as high as 64.78%. This phenomenon is attributable to the formation of different crystal phases caused by different alkali metal‐modified manganese oxides.

**TABLE 1:** Surface compositions and oxidation states of Mn and O species over the as‐prepared catalysts derived from XPS analyses.

Catalysts	Mole fraction					
	Mn			O		
	Mn^4+^	Mn^3+^	Mn^2+^	O‐I	O‐II	O‐III
Mn_2_O_3_	19.39%	42.11%	38.50%	11.18%	30.00%	58.82%
Li_0.3_MnO_δ_	32.10%	46.90%	20.99%	10.00%	37.37%	52.63%
Na_0.3_MnO_δ_	55.40%	32.21%	12.40%	8.54%	41.23%	50.25%
K_0.3_MnO_δ_	64.07%	29.44%	6.49%	6.76%	48.31%	44.93%
Rb_0.3_MnO_δ_	57.31%	30.38%	12.31%	4.78%	59.52%	35.71%
Cs_0.3_MnO_δ_	64.78%	29.13%	6.09%	4.50%	64.52%	30.97%

Upon analysis of the O 1s spectrum in Figure 3h through peak fitting, three distinct oxygen species are identified and classified as O‐I, O‐II, and O‐III, respectively. The O‐I species observed at a binding energy of approximately 533.5 eV corresponds to the surface‐adsorbed OH/H_2_O or physically adsorbed oxygen. The O‐II species observed at a binding energy of approximately 532 eV are surface‐absorbed oxygen. The O‐III species observed at a binding energy of approximately 530.5–528.8 eV belong to the lattice oxygen ions.^[57]^ The O‐II species of the alkali metal‐modified Mn‐based oxide catalyst increased significantly. Based on the XPS characterization results of Mn 2p and Mn 3s, the crystal phase formed by alkali metal modification of manganese oxide significantly increases the average oxidation state of Mn^n+^, with a higher abundance of Mn^4+^. On the catalyst surface, the O_2_ molecule undergoes adsorption at adjacent Mn^4+^ ions in a bidentate configuration, leading to dissociation into two active O* species and thereby achieving a minimal reaction potential barrier.^[58]^ Therefore, an elevation in the surface Mn^4+^ concentration contributes to enrichment of reactive oxygen species. The Cs_0.3_MnO_δ_ catalyst has a higher O‐II species abundance (64.52%), which aligns well with the findings from the O_2_‐TPD characterization (Table 1).

#### 3.2.3 NO‐TPO of the as‐prepared catalysts

**FIGURE 4:**
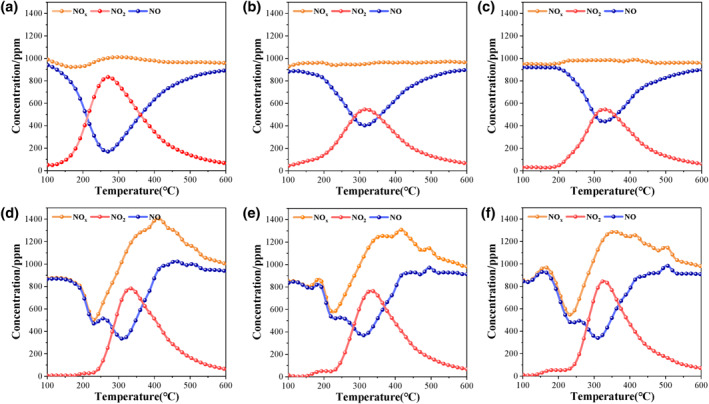
NO, NO_2_ and NO_x_ concentrations over as‐prepared catalysts. (a) Mn_2_O_3_; (b) Li_0.__3_MnO_δ_; (c) Na_0.3_MnO_δ_; (d) K_0.3_MnO_δ_; (e) Rb_0.3_MnO_δ_; (f) Cs_0.3_MnO_δ_, the reaction gas: 1000 ppm NO + 5% O_2_ + Ar balance.

The NO_2_‐assist mechanism holds a pivotal position in the soot combustion process. NO_2_ with strong oxidizability is more likely to oxidize soot. Therefore, the ability of the catalyst to convert nitrogen oxides will significantly affect its catalytic activity. The conversion ability of the prepared catalysts to nitrogen oxides was investigated by NO‐TPO. To better elucidate the role of catalysts in the conversion of NO_x_, Figure S2 shows the result of NO‐TPO without the addition of catalysts. The result shows that as the temperature increases, the concentrations of NO and NO_2_ remain constant, with only about 100 ppm of NO_2_ generated. With the participation of catalysts, the reaction of NO conversion to NO_2_ was significantly promoted (Figure 4). Comparative analysis shows that the peak concentration of NO_2_ converted by the Li_0.3_MnO_δ_ and Na_0.3_MnO_δ_ catalysts is lower than that of the other catalysts. Although the Mn_2_O_3_ catalyst has good conversion ability for nitrogen oxides, the conversion temperature is too low (less than 300°C). At the *T*_50_ temperature of the Mn_2_O_3_ catalyst, the concentration of NO_2_ was greatly reduced, and the *T*_50_ temperature and the peak concentration of NO_2_ were missed. In Figure 4d–f, it can be observed that the concentration of nitrogen oxides showed an obvious wave trough at approximately 230°C during catalysis over the K_0.3_MnO_δ_, Rb_0.3_MnO_δ_ and Cs_0.3_MnO_δ_ catalysts, indicating the adsorption and storage process of nitrogen oxides at low temperatures. With increasing temperature, the NO oxidation ability of the catalysts increases, and the stored nitrogen oxides are released as NO_2_. At approximately 330°C, the NO_2_ concentration attains its maximum, coinciding with the *T*_50_ of the catalysts, thereby facilitating the effective involvement of NO_2_ in the soot catalytic combustion process.

### 3.3 Catalytic performance of the as‐prepared catalysts

#### 3.3.1 Catalytic activities for soot combustion

The performance of the synthesized catalysts is presented in Table 2. To compare the performance of each catalyst more directly, the combustion reaction of pure soot particles without the catalyst was carried out under the same reaction conditions. The results show that all the prepared catalysts can significantly reduce the temperature of soot combustion and greatly improve Sco_2_^m^/%. The activity order (*T*_50_) of different single‐metal oxide catalysts was Mn_2_O_3_> CeO_2_> Co_3_O_4_> Fe_2_O_3_> CuO > La_2_O_3_> NiO. Among them, the hollow nanotubular Mn_2_O_3_ catalyst possessed the best catalytic performance.

**TABLE 2:** Catalytic activity of the as‐prepared catalysts (The reaction gas: 2000 ppm NO + 10% O_2_ + Ar balance).

Catalysts	*T*_10_/^o^C	*T*_50_/^o^C	*T*_90_/^o^C	Sco_2_^m^/%
Pure soot	457	552	594	41.0%
Mn_2_O_3_	315	380	420	97.9%
Fe_2_O_3_	359	428	463	92.3%
Co_3_O_4_	327	394	443	99.3%
NiO	388	466	500	97.1%
CuO	365	428	449	98.2%
La_2_O_3_	351	432	471	90.1%
CeO_2_	330	392	432	85.7%
Li_0.3_MnO_δ_	329	398	433	88.7%
Na_0.3_MnO_δ_	299	347	378	97.7%
K_0.3_MnO_δ_	274	324	352	98.4%
Rb_0.3_MnO_δ_	291	338	369	95.3%
Cs_0.3_MnO_δ_	267	324	360	97.8%

Based on the above catalytic activities of different single‐metal oxide catalysts, alkali metals were used to modify the Mn‐based oxides with hollow nanotubular structures. The catalytic performances of alkali metal‐modified Mn‐based oxides were significantly different (Table 2). The activity order (*T*_50_) is as follows: Cs_0.3_MnO_δ_> K_0.3_MnO_δ_> Rb_0.3_MnO_δ_> Na_0.3_MnO_δ_> Li_0.3_MnO_δ_. The hollow nanotubular Cs_0.3_MnO_δ_ catalyst exhibited the best catalytic performance, with *T*_10_, *T*_50_, *T*_90_, and Sco_2_^m^/% of 267°C, 324°C, 360°C and 97.8%, respectively.

#### 3.3.2 Stability of the Cs_0.3_MnO_δ_ catalyst

**FIGURE 5:**
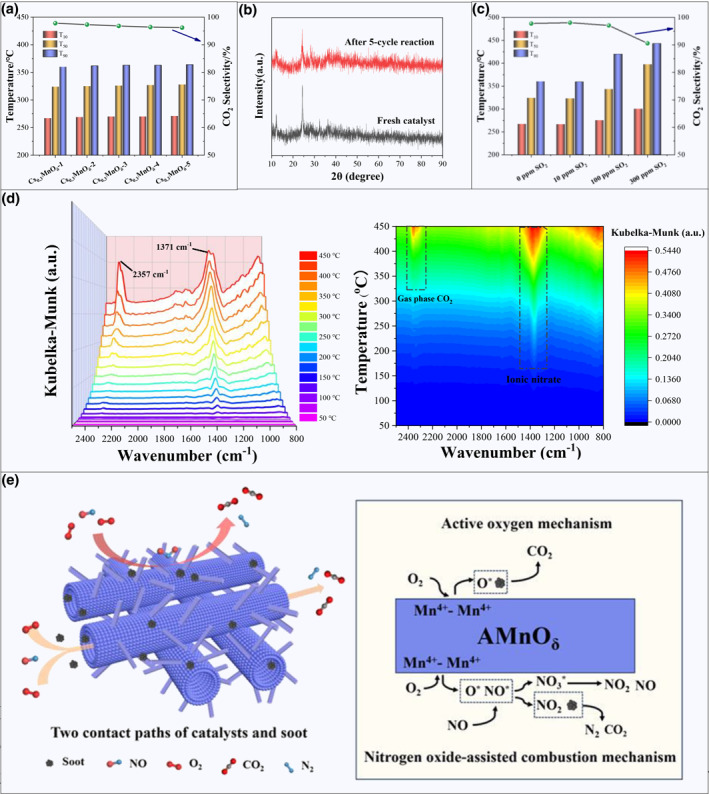
Stability of Cs_0.3_MnO_δ_ catalyst, the reaction gas: 1000 ppm NO + 5% O_2_+Ar balance (a); XRD patterns of Cs_0.3_MnO_δ_ catalyst (b); Tolerance of sulfur dioxide, the reaction gas: 2000 ppm NO + 10% O_2_ + 10/100/300ppm SO_2_ + Ar balance (c); In situ DRIFTS spectra of Cs_0.3_MnO_δ_ catalyst, the reaction gas: 1000 ppm NO + 5% O_2_ + Ar balance (d); The reaction mechanism of catalytic soot combustion over Cs_0.3_MnO_δ_ catalyst (e).

Stability of catalysts is critical in industrial applications. Therefore, under the same activity evaluation conditions, the Cs_0.3_MnO_δ_ catalyst was cycle evaluated (Figure 5a). The activity evaluation results showed that the Cs_0.3_MnO_δ_ catalyst possessed excellent stability. In the five cycle activity evaluations, *T*_10_, *T*_50_, and *T*_90_ were 269 ± 2°C, 326 ± 2°C, and 362 ± 2°C, respectively, and the CO_2_ selectivity remained above 95% throughout the cycle tests, indicating the superior stability of the catalyst. To better study the effect of cyclic testing on the crystal structure of the Cs_0.3_MnO_δ_ catalyst, the XRD patterns of the Cs_0.3_MnO_δ_ catalyst after cyclic testing and the fresh catalyst were compared. The characteristic peak of the catalyst remained basically unchanged, indicating that the Cs_0.3_MnO_δ_ catalyst possesses a highly stable crystal structure (Figure 5b).

#### 3.3.3 Sulfur resistance of the Cs_0.3_MnO_δ_ catalyst

As is well known, SO_2_ is toxic to most catalysts, and diesel engine exhaust often contains a small amount of SO_2_, which may lead to catalyst sulfur poisoning and decreased activity. Therefore, the sulfur resistance of catalysts is one of the important evaluation criteria for their industrialization. We evaluated the sulfur resistance performance of Cs_0.3_MnO_δ_ catalyst in different concentrations of SO_2_. As shown in the Figure 5c, the activty of the Cs_0.3_MnO_δ_ catalyst is basically consistent with the activity results in a sulfur free environment when the catalyst was tested in the activity evaluation closest to the actual sulfur content in diesel engine exhaust (10 ppm SO_2_), which demonstrates excellent sulfur resistance performance. However, with the increase in SO_2_ concentration, the activity of Cs_0.3_MnO_δ_ catalyst shows a decreasing trend, especially when the *T*_90_ temperature increased significantly.

### 3.4 Possible reaction mechanism of soot combustion on the as‐prepared catalysts

In the prepared different single‐metal oxide catalysts with fibrous structures, the Mn_2_O_3_ catalyst shows better catalytic activity because of its excellent redox ability. After further modification with alkali metals, the crystal form, morphology, and redox properties of the catalysts changed significantly. The characterization results show that alkali metal modification exerts a significant effect on the amount of active oxygen, redox ability, and nitrogen oxide conversion ability of the catalysts. The differing electronegativities, alkalinities, and electron‐donating abilities of different alkali metals result in the differing physicochemical properties of the as‐prepared catalysts. The results of XPS and H_2_‐TPR characterization show that alkali metal doping can effectively affect the electronic configuration of manganese ions in catalysts, which not only improves the oxidation state of manganese species but also improves the oxidation ability of the catalyst itself. The increase in the abundance of Mn^4+^ species is beneficial for adsorbing and activating O_2_ molecules. At the same time, the valence state change of Mn^n+^ is easier than that of the pure manganese phase, and the rapid transition of the valence state is conducive to forming reactive oxygen species. The characterization results of O_2_‐TPD, soot‐TPR, and XPS show that alkali metals as electron donors can cause the manganese oxide to form more oxygen defects, enhance the mobility of oxygen species, and facilitate the generation, activation and transport of active oxygen species. The reactive oxygen species of Mn‐based oxide catalysts modified with alkali metals increased significantly. Due to its strong oxidizing properties, NO_2_ can promote the reaction process of soot catalytic combustion. At the same time, alkali metals are highly favorable for the adsorption of acidic nitrogen oxide gases because of their alkalinity. The characterization results of NO‐TPO indicate that the prepared K_0.3_MnO_δ_, Rb_0.3_MnO_δ_, and Cs_0.3_MnO_δ_ catalysts all have good NO oxidation abilities. In order to better explore the contribution of nitrogen oxides and nitrate species formed in the catalytic soot combustion reaction, the Cs_0.3_MnO_δ_ catalyst was characterized using in situ DRIFTS. As shown in Figure 5d, as the operating temperature increases, it can be observed that the absorption peaks at 2357 and 1371 cm^−1^ gradually increase, and the two absorption peaks correspond to gas phase CO_2_ and ion nitrate species, respectively.[^59^, ^60^] The enhancement of the absorption peak of gas phase CO_2_ indicates that the rate of CO_2_ formation from soot catalytic combustion increases with the increase of temperature. The enhancement of the absorption peak of ionic nitrate species indicates that ion nitrate species are the main nitrate species in the catalytic combustion of soot. Combined with the characterization results of in situ DRIFTS and NO‐TPO, it can be determined that NO forms two nitrogen‐containing compounds, NO_2_ and ionic nitrate species, under the action of the catalysts. The participation of NO_2_ can accelerate the catalytic combustion of soot. Among the characterization results of the alkali metal‐modified Mn‐based oxide catalysts, the Cs_0.3_MnO_δ_ catalyst has the highest oxidation state of Mn species, the strongest redox ability, the most active oxygen species, and excellent storage and conversion ability for nitrogen oxides.

Combining the above characterization and catalytic performance results, it is considered that the catalytic soot combustion process of the Cs_0.3_MnO_δ_ catalyst is completed under the combined action of the reactive oxygen reaction mechanism and the nitrogen oxide‐assisted combustion mechanism (Figure 5e). First, O_2_ is adsorbed on the surface of the active centers, and under the activation of high‐valent Mn^4+^–Mn^4+^, it is activated into reactive oxygen species (O^−^ and O_2_^−^) through the MvK mechanism. A portion of the generated reactive oxygen species migrate to the soot surface and directly oxidize the soot to produce CO_2_. The other portion generates NO_2_ and ionic nitrate species through the L–H mechanism, utilizing the adsorbed NO* produced by the activation of NO on the catalyst surface. The soot reacts with the more oxidizing NO_2_ and nitrate species to generate CO_2_ and N_2_, and some nitrate species are further decomposed into NO and NO_2_ under high‐temperature conditions.

Because of the special hollow nanotube structure of Cs_0.3_MnO_δ_ catalyst, the contact efficiency between soot and the catalyst was significantly enhanced. The soot can penetrate the tubular structure of the catalyst under the driving of the reaction airflow, greatly improving the effective catalytic contact area of the catalyst and exposing more active sites. Meanwhile, the dense distribution of nanoneedle structures on the catalyst surface promotes effective interception of soot particles, allowing sufficient contact between soot and catalyst for catalytic combustion.

## 4 CONCLUSION

In this study, different single‐metal oxide catalysts with fibrous structures and alkali metal‐modified hollow nanotubular Mn‐based oxide catalysts were prepared using a centrifugal spinning method using metal acetate and nitrate as raw materials and PVP as a template. Among them, the Mn_2_O_3_ catalyst has the best catalytic performance among the prepared single‐metal oxide catalysts. After modification with different alkali metals, the catalytic performance of the Mn‐based oxide catalysts was significantly improved. Among them, the Cs_0.3_MnO_δ_ catalyst shows the best catalytic performance because of its highest abundance of active oxygen species, Mn^4+^ species, and excellent nitrogen oxide conversion ability, combined with the structural effect caused by the special morphology of the hollow nanotube structure. The as‐prepared catalysts have the advantages of high production efficiency, good catalytic activity, strong stability, good sulfur resistance, and excellent application potential. Additionally, this study provides more references for alkali metal modification and Mn‐based oxide catalysts in producing effective catalytic soot combustion and other oxidation reactions.

## CONFLICT OF INTEREST STATEMENT

The authors declare no conflicts of interest.

## ETHICS STATEMENT

No animal or human experiments were involved in this study.

## Supporting information

Supporting Information S1

## Data Availability

The data that support the findings of this study are available from the corresponding author upon reasonable request.

## References

[1] E. Long , C. Carlsten , Part. Fibre Toxicol. 2022, 19, 11. 35139881 10.1186/s12989-022-00450-5 PMC8827176

[2] M. H. G. Andersen , M. Frederiksen , A. T. Saber , R. S. Wils , A. S. Fonseca , I. K. Koponen , S. Johannesson , M. Roursgaard , S. Loft , P. Møller , U. Vogel , Part. Fibre Toxicol. 2019, 16, 21. 31182122 10.1186/s12989-019-0306-4 PMC6558821

[3] N. E. Alexis , C. Carlsten , Int. Immunopharmacol. 2014, 23, 347. 25194677 10.1016/j.intimp.2014.08.009

[4] R. K. Robinson , M. A. Birrell , J. J. Adcock , M. A. Wortley , E. D. Dubuis , S. Chen , C. M. McGilvery , S. Hu , M. S. P. Shaffer , S. J. Bonvini , S. A. Maher , I. S. Mudway , A. E. Porter , C. Carlsten , T. D. Tetley , M. G. Belvisi , J. Allergy Clin. Immunol. 2018, 141, 1074. 28532657 10.1016/j.jaci.2017.04.038 PMC5840514

[5] S. Gao , D. Yu , S. Zhou , C. Zhang , L. Wang , X. Fan , X. Yu , Z. Zhao , J. Mater. Chem. A 2023, 11, 19210.

[6] X. Wang , Y. Wang , Y. Bai , P. Wang , Y. Zhao , J. Energy Inst. 2019, 92, 1864.

[7] S. J. Wilson , M. R. Miller , D. E. Newby , Antioxid. Redox Signaling 2018, 28, 819. 10.1089/ars.2017.7174 28540736

[8] H. Reis , C. Reis , A. Sharip , W. Reis , Y. Zhao , R. Sinclair , L. Beeson , Environ. Int. 2018, 114, 252. 29524921 10.1016/j.envint.2018.02.042

[9] C. Zhang , D. Yu , C. Peng , L. Wang , X. Yu , Y. Wei , J. Liu , Z. Zhao , Appl. Catal. B Environ. 2022, 319, 121946.

[10] B. A. A. L. van Setten , M. Makkee , J. A. Moulijn , Catal. Rev. 2001, 43, 489.

[11] J. C. Clerc , Appl. Catal. B Environ. 1996, 10, 99.

[12] Y. Yang , D. Zhao , Z. Gao , Y. Tian , T. Ding , J. Zhang , Z. Jiang , X. Li , Appl. Catal. B Environ. 2021, 286, 119932.

[13] Y. Wei , P. Zhang , J. Xiong , Q. Yu , Q. Wu , Z. Zhao , J. Liu , Environ. Sci. Technol. 2020, 54, 6947. 32374163 10.1021/acs.est.0c00752

[14] B. Cui , K. Zhou , M. Hu , T. Zhao , Y. Liu , Y. Li , Z. Shao , M. Zhao , Chem. Eng. J. 2023, 461, 142107.

[15] Y. Wei , Z. Zhao , J. Liu , S. Liu , C. Xu , A. Duan , G. Jiang , J. Catal. 2014, 317, 62.

[16] L. Wang , X. Yu , Y. Wei , J. Liu , Z. Zhao , J. Rare Earths 2021, 39, 1151.

[17] A. Rangaswamy , P. Sudarsanam , B. M. Reddy , J. Rare Earths 2015, 33, 1162.

[18] W. Zhan , Y. Guo , X. Gong , Y. Guo , Y. Wang , G. Lu , Chin. J. Catal. 2014, 35, 1238.

[19] F. Ji , Y. Men , J. Wang , Y. Sun , Z. Wang , B. Zhao , X. Tao , G. Xu , Appl. Catal. B Environ. 2019, 242, 227.

[20] N. S. Portillo‐Vélez , R. Zanella , Chem. Eng. J. 2020, 385, 123848.

[21] C. Cao , H. Yang , J. Xiao , X. Yang , B. Ren , L. Xu , G. Liu , X. Li , Fuel 2021, 305, 121446.

[22] D. Yu , Y. Ren , X. Yu , X. Fan , L. Wang , R. Wang , Z. Zhao , K. Cheng , Y. Chen , Z. Sojka , A. Kotarba , Y. Wei , J. Liu , Appl. Catal. B Environ. 2021, 285, 119779.

[23] I. Atribak , A. Bueno‐López , A. García‐García , P. Navarro , D. Frías , M. Montes , Appl. Catal. B Environ. 2010, 93, 267.

[24] D. Yu , X. Yu , C. Zhang , L. Wang , X. Fan , Z. Zhao , Y. Wei , J. Liu , J. Gryboś , B. Leszczyński , A. Wach , D. Wierzbicki , A. Kotarba , Z. Sojka , Appl. Catal. B Environ. 2023, 338, 123022.

[25] C. Peng , D. Yu , C. Zhang , M. Chen , L. Wang , X. Yu , X. Fan , Z. Zhao , K. Cheng , Y. Chen , Y. Wei , J. Liu , J. Environ. Sci. 2023, 125, 82. 10.1016/j.jes.2021.10.029 36375963

[26] D. Yu , L. Wang , C. Zhang , C. Peng , X. Yu , X. Fan , B. Liu , K. Li , Z. Li , Y. Wei , J. Liu , Z. Zhao , ACS Catal. 2022, 12, 15056.

[27] C. Zhang , S. Gao , S. Zhou , D. Yu , L. Wang , X. Fan , X. Yu , B. Liu , Z. Zhao , Appl. Catal. B Environ. 2024, 355, 124169.

[28] Y. Tsai , N. Huy , J. Lee , Y. Lin , K. Lin , Chem. Eng. J. 2020, 395, 124939.

[29] S. Zhou , L. Wang , S. Gao , X. Chen , C. Zhang , D. Yu , X. Fan , X. Yu , Z. Zhao , ACS Catal. 2024, 14, 6062.

[30] C. Zhang , D. Yu , L. Wang , X. Yu , Z. Zhao , Sci. Sin. Chim. 2023, 53, 1636.

[31] F. Fang , N. Feng , P. Zhao , C. Chen , X. Li , J. Meng , G. Liu , L. Chen , H. Wan , G. Guan , Chem. Eng. J. 2019, 372, 752.

[32] H. Liang , B. Jin , M. Li , X. Yuan , J. Wan , W. Liu , X. Wu , S. Liu , Appl. Catal. B Environ. 2021, 294, 120271.

[33] C. Lee , J. I. Park , Y. G. Shul , H. Einaga , Y. Teraoka , Appl. Catal. B Environ. 2015, 174−175, 185.

[34] R. Liu , L. Hou , G. Yue , H. Li , J. Zhang , J. Liu , B. Miao , N. Wang , J. Bai , Z. Cui , T. Liu , Y. Zhao , Adv. Fiber Mater. 2022, 4, 604.

[35] X. Zhang , Z. Ru , Y. Sun , M. Zhang , J. Wang , M. Ge , H. Liu , S. Wu , C. Cao , X. Ren , J. Mi , Y. Feng , J. Clean. Prod. 2022, 378, 134567.

[36] T. Boningari , D. Pappas , P. Smirniotis , J. Catal. 2018, 365, 320.

[37] Z. Zhang , R. Li , M. Wang , Y. Li , Y. Tong , P. Yang , Y. Zhu , Appl. Catal. B Environ. 2021, 282, 119542.

[38] A. Vakhrushev , T. Boitsova , J. Porous Mater. 2021, 28, 1023.

[39] J. Ko , B. Lee , Y. Chung , C. Park , Green Chem. 2015, 17, 4167.

[40] H. Li , N. Yuan , J. Qian , B. Pan , Environ. Sci. Technol. 2022, 56, 4498. 35297618 10.1021/acs.est.1c08790

[41] Z. Chen , Y. Ma , B. Geng , M. Wang , X. Sun , J. Alloys Compd. 2017, 700, 113.

[42] T. Hong , Z. Liu , X. Zheng , J. Zhang , L. Yan , Appl. Catal. B Environ. 2017, 202, 454.

[43] F. Yuan , Y. Ni , L. Zhang , S. Yuan , J. Wei , J. Mater. Chem. A 2013, 1, 8438.

[44] Y. Wu , J. Li , M. Lv , X. Zhang , R. Gao , C. Guo , X. Cheng , X. Zhou , Y. Xu , S. Gao , Z. Major , L. Huo , J. Hazard Mater. 2023, 442, 130041. 36166911 10.1016/j.jhazmat.2022.130041

[45] Y. Li , L. Yue , L. Yue , L. Jia , J. Liu , K. Xie , X. Yang , Y. Zhang , Sens. Actuators, B 2023, 378, 133125.

[46] H. Zheng , W. Liao , J. Ding , F. Xu , A. Jia , W. Huang , Z. Zhang , ACS Catal. 2022, 12, 15451.

[47] D. Liu , S. Sun , J. Yu , Chem. Eng. J. 2019, 377, 119825.

[48] D. Yu , C. Peng , Y. Ren , L. Wang , C. Zhang , X. Fan , X. Yu , Z. Zhao , Appl. Catal. B Environ. 2024, 344, 123614.

[49] Y. Zhang , F. Tao , S. Cao , K. Yin , X. Chang , R. Fan , C. Fan , L. Dong , Y. Yin , X. Chen , Sol. Energy Mater. Sol. Cells 2019, 200, 110043.

[50] P. Legutko , J. Gryboś , M. Fedyna , J. Janas , A. Wach , J. Szlachetko , A. Adamski , X. Yu , Z. Zhao , A. Kotarba , Z. Sojka , Catalysts 2020, 10, 1390.

[51] Y. Wang , K. Liu , J. Wu , Z. Hu , L. Huang , J. Zhou , T. Ishihara , L. Guo , ACS Catal. 2020, 10, 10021.

[52] Q. Sun , J. He , X. Li , T. Lu , W. Si , F. Zhao , K. Wang , C. Huang , Chem. Eng. J. 2022, 432, 134402.

[53] Q. Yang , X. Wang , H. Wang , X. Li , Q. Li , Y. Wu , Y. Peng , Y. Ma , J. Li , Appl. Catal. B Environ. 2023, 320, 121993.

[54] C. Zhang , D. Yu , C. Peng , L. Wang , X. Fan , X. Yu , Z. Zhao , Processes 2021, 9, 1149.

[55] M. Zhao , L. Deng , J. Liu , Y. Li , J. Liu , Z. Duan , J. Xiong , Z. Zhao , Y. Wei , W. Song , Y. Sun , ACS Catal. 2019, 9, 7548.

[56] F. Fang , P. Zhao , N. Feng , C. Chen , X. Li , G. Liu , H. Wan , G. Guan , Catal. Sci. Technol. 2019, 9, 4938.

[57] J. Kong , S. Song , W. Zhao , Z. Yu , Z. Xiang , G. Li , W. Zhang , T. An , Appl. Catal. B Environ. 2023, 339, 123118.

[58] C. Peng , Y. Ren , D. Yu , L. Wang , C. Zhang , X. Fan , X. Yu , Z. Zhao , Y. Wei , J. Liu , Nano Res. 2023, 16, 6187.

[59] J. Liu , Y. Du , J. Liu , Z. Zhao , K. Cheng , Y. Chen , Y. Wei , W. Song , X. Zhang , Appl. Catal. B Environ. 2017, 203, 704.

[60] Z. Zhang , Y. Zhang , Q. Su , Z. Wang , Q. Li , X. Gao , Environ. Sci. Technol. 2010, 44, 8254. 20923141 10.1021/es102363f

