# Supplementary material for: Facile preparation of alkali metal‐modified hollow nanotubular manganese‐based oxide catalysts and their excellent catalytic soot combustion performance
Source: Smart Mol. 2024 Jul 15;3(1):e20240022. doi: 10.1002/smo.20240022 (PMC12117889; doi:10.1002/smo.20240022)
Supplement: Supplementary file 1 — Supporting Information S1 [file SMO2-3-e20240022-s001.docx]

**Supporting information for**

**Facile preparation of** **alkali metal-modified** **hollow nanotubular manganese-based oxide catalysts and their excellent catalytic soot combustion performance**

Chunlei Zhang^1^, Siyu Gao^2^, Xinyu Chen^2^, Di Yu^1^, Lanyi Wang^2^, Xiaoqiang Fan^2^, Ying Cheng^3^, Xuehua Yu^2^*, Zhen Zhao^1,^^2^*

^1^ State Key Laboratory of Heavy Oil Processing, China University of Petroleum, 18# Fuxue Road, Chang Ping, Beijing 102249, China

^2^ Institute of Catalysis for Energy and Environment, College of Chemistry and Chemical Engineering, Shenyang Normal University, Shenyang, Liaoning, 110034, China

^3.^Department of Environmental Engineering, Hebei University of Environmental Engineering, Qinhuangdao, Hebei, 066102, China

**Catalyst characterization procedures:**

The XRD pattern was obtained using an X-ray diffractometer (Ultima IV, Rigaku). The radiation source was Cu Kα and nickel filters. The pattern acquisition range was 2θ = 10°–90°. The surface morphology of the catalysts was observed using either a Hitachi SU8010N or Hitachi S4800 scanning electron microscope. The textural properties of the catalysts were determined using a Micromeritics TriStar II: 3020 analyzer. The H_2_-TPR curve was obtained using a TP-5080 adsorption instrument, Tianjin. A 100 mg sample was pretreated in flowing nitrogen at 300 °C for 1 h and then reduced to room temperature. The catalysts were purged with 10% H_2_–Ar mixed gas, and the temperature was increased from room temperature to 800 °C at a rate of 10 °C·min^−1^. The O_2_-TPD curve was also obtained using the TP-5080 adsorption instrument, Tianjin. The 100 mg samples were pretreated in flowing oxygen at 300 °C for 1 h, then reduced to room temperature and purged for another 30 min. The catalysts were then purged with He and heated from room temperature to 800 °C at a rate of 10 °C·min^−1^. The soot-TPR curve was obtained using an Agilent 7890B gas chromatograph. In an Ar atmosphere at a flow rate of 50 mL·min^−1^, the loose contact of 10 mg soot and 100 mg catalyst was heated from 100 °C to 800 °C at a rate of 4 °C·min^−1^. XPS spectra were obtained using a Thermo ESCALAB 250 Xi instrument. NO-TPO measurements were performed on a MultiGas 6030 Fourier transform infrared spectrometer. The total flow rate of the reaction gas was 100 mL·min^−1^, the composition of the reaction gas was 1000 ppm NO, 5% O_2_, and the balance was Ar. DRIFTS were characterized on a Vertex 80v spectrometer (Bruker). The mixture of catalyst and soot with the ratio of 10:1 was pretreated at 300 °C for 30 min under argon protection and then cooled to room temperature. Subsequently, the gas flow was switched to the same gas component as that used to evaluate the catalytic activity, and the spectrum was collected at a heating rate of 5 ^o^C·min^-1^ in the temperature range of 50-400 °C in the range of 800-2500 cm^-1^.

**Catalytic activity evaluation procedures:**

The catalytic performance of the catalysts was evaluated using the temperature-programmed oxidation method. The soot used were Printx-U particles (Φ=25 nm) purchased from Degussa. A mixture of 100 mg catalyst and 10 mg soot in loose contact was placed in the fixed-bed quartz tube reactor (Φ=8 mm). The total flow rate of the reaction gas was 50 mL·min^−1^, the gas composition was 2000 ppm NO, 10% O_2_, and the balance was Ar. The heating program started at 100 °C, and the heating rate was 2 °C·min^−1^ until the soot was burned out. The composition of CO and CO_2_ in the outlet gas was analyzed using a gas chromatograph (Agilent 7890B) and a hydrogen flame ionization detector. The temperatures (*T*_10_, *T*_50_, and *T*_90_) when the soot particle combustion conversion rate reaches 10%, 50%, and 90% are used as the basis for evaluating the catalyst activity, and the CO_2_ selectivity is calculated by the following Equation 1:

$S_{{CO}_{2}}^{m}=\frac{{{CO}_{2}}_{out}^{max}}{{{CO}_{2}}_{out}^{max}+{CO}_{out}^{max}}\times100\%$ (Equation 1)

where $[\text{C}\text{O}_{2}]_{\text{out}}^{\text{max}}$ and $[\text{CO}]_{\text{out}}^{\text{max}}$ are the concentrations of CO_2_ and CO, respectively, in the outlet gas at the temperature (*T*_m_) with the fastest burning rate of soot, and *S*_CO2_^m^ is the selectivity of CO_2_ at *T*_m_.

The stability of the catalysts was evaluated by the catalytic activity of the catalysts in five consecutive reaction cycles. When investigating the sulfur resistance of the catalysts, different concentrations of SO_2_ (10 ppm, 100 ppm or 300 ppm) were added to the reaction gas for activity evaluation.

**Table S1** Expression and preparation raw materials of different single metal oxide catalysts with fibrous shape

| Catalysts | M(NO_3_)_x_·xH_2_O /g  (CH_3_COO)_x_·xH_2_O M/g | PVP/g | CH_3_CH_2_OH /mL | H_2_O/mL | CH_3_COOH /mL |
| --- | --- | --- | --- | --- | --- |
| Mn_2_O_3_ | 2.4509 | 1.9 | 2 | 7.5 | 0.5 |
| Fe_2_O_3_ | 4.04 | 2 | 3.5 | 6 | 0.5 |
| Co_3_O_4_ | 2.4908 | 1.75 | 2 | 7.5 | 0.5 |
| NiO | 2.9079 | 2 | 5.5 | 4 | 0.5 |
| CuO | 2.416 | 5 | 4.5 | 5 | 0.5 |
| La_2_O_3_ | 4.33 | 1.6 | 2 | 8 | 0 |
| CeO_2_ | 4.3422 | 1.625 | 2 | 8 | 0 |

**Table S2** Expression and preparation raw materials of alkali metal-modified hollow nanotubular Mn-based oxide catalysts

| Catalysts | CH_3_COOA·xH_2_O/g | Mn(CH_3_COO)_2_·4H_2_O | PVP/g | CH_3_CH_2_OH /mL | H_2_O/mL | CH_3_COOH /mL |
| --- | --- | --- | --- | --- | --- | --- |
| Li_0.3_MnO_δ_ | 0.6121 | 4.9018 | 2 | 2 | 7.5 | 0.5 |
| Na_0.3_MnO_δ_ | 0.4922 | 4.9018 | 2 | 2 | 7.5 | 0.5 |
| K_0.3_MnO_δ_ | 0.5888 | 4.9018 | 2 | 2 | 7.5 | 0.5 |
| Rb_0.3_MnO_δ_ | 0.8671 | 4.9018 | 2 | 2 | 7.5 | 0.5 |
| Cs_0.3_MnO_δ_ | 1.1517 | 4.9018 | 2 | 2 | 7.5 | 0.5 |

**Table S3.** Texture properties of as-prepared catalysts

| Catalysts | BET/(m²/g)^a^ | Pore volume /(cm³/g) ^b^ | Pore size/(nm) ^c^ |
| --- | --- | --- | --- |
| Mn_2_O_3_ | 5.1 | 0.032 | 33.8 |
| Fe_2_O_3_ | 13.0 | 0.043 | 14.9 |
| Co_3_O_4_ | 10.6 | 0.066 | 23.8 |
| NiO | 14.0 | 0.088 | 21.3 |
| CuO | 1.8 | 0.007 | 28.3 |
| La_2_O_3_ | 24.4 | 0.108 | 15.9 |
| CeO_2_ | 27.4 | 0.040 | 5.7 |
| Li_0.3_MnO_δ_ | 9.1 | 0.063 | 24.8 |
| Na_0.3_MnO_δ_ | 6.5 | 0.033 | 23.7 |
| K_0.3_MnO_δ_ | 10.2 | 0.056 | 34.0 |
| Rb_0.3_MnO_δ_ | 5.9 | 0.025 | 16.1 |
| Cs_0.3_MnO_δ_ | 2.6 | 0.013 | 33.5 |

a: Calculated by BET method; b: The total pore volume of pores with a diameter of 1.7-300 nm was calculated by BJH method; c: The average pore size was calculated by BJH method.


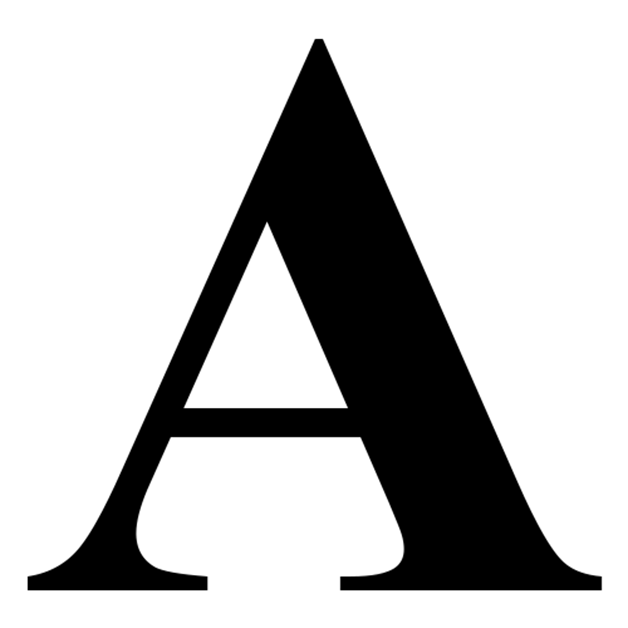

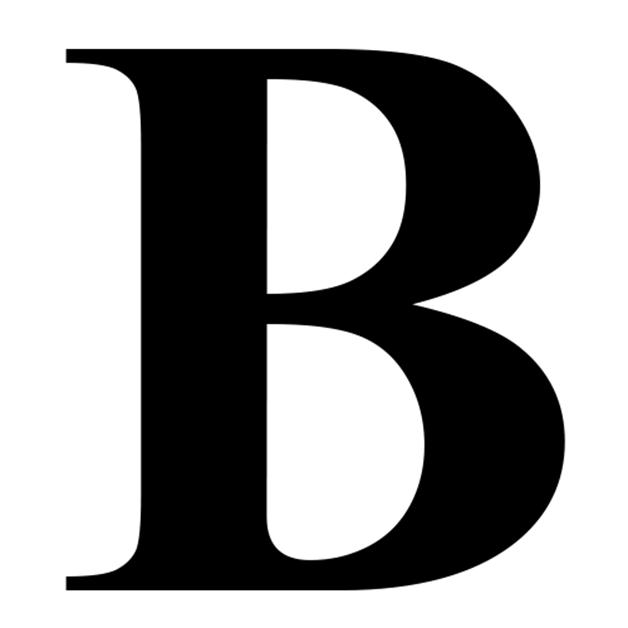

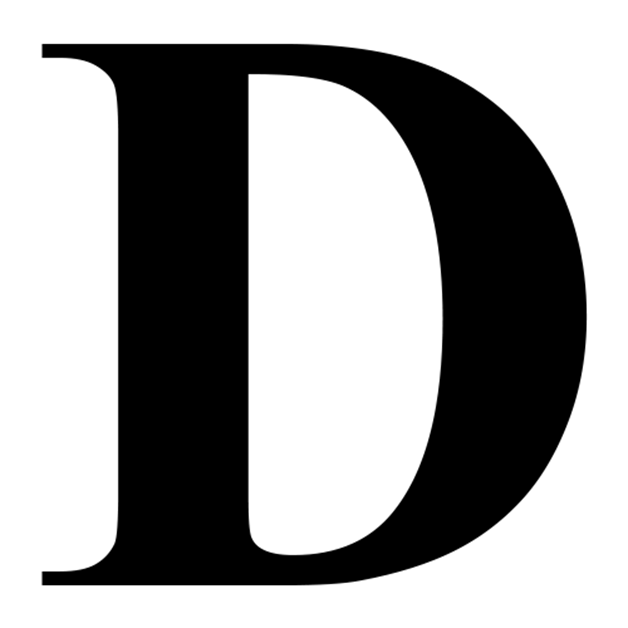

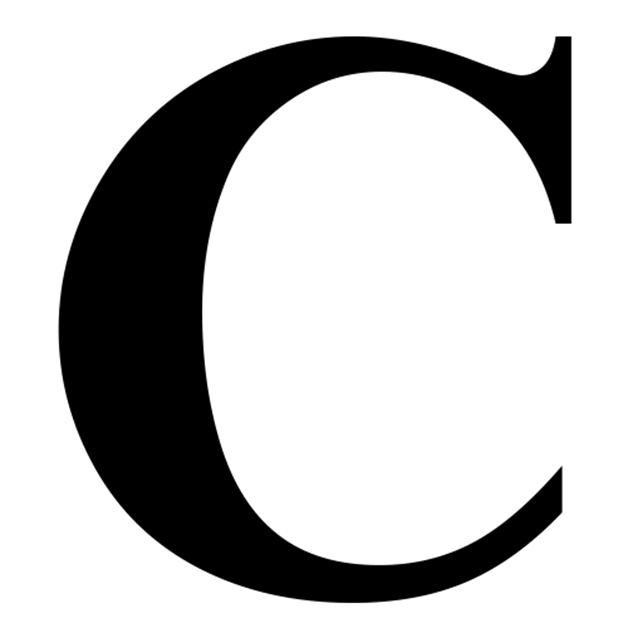


**Figure S1** N_2_ adsorption-desorption isotherms (A) and BJH pore size distribution (B) of different single metal oxide catalysts with fibrous shape; N_2_ adsorption-desorption isotherms (C) and BJH pore size distribution (D) of alkali metal-modified hollow nanotubular Mn-based oxide catalysts

(A,B: a: Mn_2_O_3_, b: Fe_2_O_3_, c: Co_3_O_4_, d: NiO, e: CuO, f: La_2_O_3_, g: CeO_2_; C,D: a: Li_0.3_MnO_δ_, b: Na_0.3_MnO_δ_, c: K_0.3_MnO_δ_, d: Rb_0.3_MnO_δ_, e: Cs_0.3_MnO_δ_)

**Figure S2** NO, NO_2_ and NO_x_ concentrations without catalysts, the reaction gas: 2000 ppm NO + 10% O_2_ + Ar balance)
